# Investigating nurses’ knowledge and attitudes about delirium in older persons: a cross-sectional study

**DOI:** 10.1186/s12912-022-01158-9

**Published:** 2023-01-11

**Authors:** Maria Papaioannou, Evridiki Papastavrou, Christiana Kouta, Haritini Tsangari, Anastasios Merkouris

**Affiliations:** 1 Cyprus University of Technology, Limassol, Cyprus; 2 University of Nicosia, Nicosia, Cyprus

**Keywords:** Acute confusion, Attitudes, Delirium, Knowledge, Nurse

## Abstract

**Introduction:**

Delirium is the most common emergency for older hospitalized patients that demands urgent treatment, otherwise it can lead to more severe health conditions. Nurses play a crucial part in diagnosing delirium and their competencies facilitate the appropriate treatment and management of the condition.

**Aim:**

This study aims to enhance the understanding of delirium care by exploring both knowledge and attitudes of nurses toward patients in acute care hospital wards and the possible association between these two variables.

**Method:**

The Nurses Knowledge of Delirium Questionnaire (NKD) and the Attitude Tool of Delirium (ATOD) that were created for the said inquiry, were disseminated to 835 nurses in the four largest Public Hospitals of the Republic. These tools focused particularly on departments with increased frequency of delirium (response rate = 67%).

**Results:**

Overall nurses have limited knowledge of acute confusion/delirium. The average of correct answers was 42.2%. Only 38% of the participants reported a correct definition of delirium, 41.6 correctly reported the tools to identify delirium and 42.5 answered correctly on the factors leading to delirium development. The results of the attitudes’ questionnaire confirmed that attitudes towards patients with delirium may not be supportive enough. A correlation between the level of nurses’ knowledge and their attitude was also found. The main factors influencing the level of knowledge and attitudes were gender, education, and workplace.

**Conclusion:**

The findings of this study are useful for the international audience since they can be used to develop and modify educational programmes in order to rectify the knowledge deficits and uninformed attitudes towards patients with delirium. The development of a valid and reliable instrument for the evaluation of attitudes will help to further assess nurses’ attitudes. Furthermore, the results are even more important and useful on a national level since there is no prior data on the subject area, making this study the first of its kind.

## Background

The term delirium (also known as acute confusion) refers to an acute confounding state, which is due to a wide range of factors including medicinal conditions, diseases, substances, drug abuse or combined causation. Delirium is a complex clinical syndrome characterized by a disturbance of people’s consciousness, attention, cognitive function, or perception, that develops within a short period of time and its symptoms can fluctuate during the day [1, 2]. Delirium is a medical emergency and is prevalent among older people. It is viewed as a medical condition that is severe, costly and recognized as an indicator of patient safety [3, 4].

Unfortunately, if delirium is not promptly diagnosed and treated, it can lead to brain damage and permanent disability. Further, it constitutes a huge financial burden as it doubles hospitalization time, the probability of death and the rate of re-admission [5]. Beyond the humanistic and the social dimension of the issue, the economic cost of delirium is enormous. International organisations [6] and scientific literature report that delirium doubles the cost of nursing care [7, 8]. This reality underlines the importance of measures towards more effective prevention [9].

The recognition and diagnosis of delirium is a difficult process that is determined mainly by the patient’s profile of symptoms. The American Psychiatric Association Diagnostic and statistical manual 2013, 5th edition [2], lists five basic criteria that characterize delirium, such as (a) disturbance in attention (b) the disturbance develops over a short period, it represents an acute change from baseline attention and awareness, and tends to fluctuate in severity during the day, (c) disturbance in cognition (d) the mentioned disturbances are not explained by a pre-existing or evolving neurocognitive disorder and (e) there is evidence that it is a direct physiological consequence of another medical condition, substance intoxication or other multiple causes. Leading scientific organizations in delirium practice, such as the American Delirium Society (ADS) and European Delirium Association (EDA) [10], through their universal approach to delirium, recommend a more comprehensive interpretation of the DSM-5 criteria for the wider prevention and detection of delirium and in turn, greater patient safety.

It is interesting to note that delirium occurs in up to 55% of older hospitalized patients [5, 11]. Concerning 31% in patients over the age of 65 [12], it is associated with an increased risk of permanent cognitive and functional impairment. At an age greater than 90 years, the prevalence and sequelae of delirium are substantial [13]. Nevertheless, if recognized early, delirium can be prevented in a large percentage of cases [14] and is often reversible with the treatment of the underlying disease [15, 16].

Risk factors of delirium include dementia, advanced age, comorbidities, decreased vision, depression, infections, and dehydration [17, 18]. In fact, evidence from systematic reviews and meta-analyses report that dementia, impaired vision, the application of a urinary catheter, low albumin levels, and longer hospital stays are associated with delirium [19]. Studies since the onset of the pandemic reveal that delirium represents a common complication of COVID-19 and a marker of severe disease course, especially in older patients with neuropsychiatric comorbidity [20].

The above discussion shows the importance of recognizing delirium. Nurses, who are the health professionals spending the most time with the patient play a key role in recognizing delirium. Unfortunately, consistent scientific data indicates that there is a low level of knowledge and subsequently reduced recognition of delirium in older patients during hospitalization [21, 22]. Parallel to the low level of knowledge, nurses’ attitudes towards older patients are particularly negative due to multiple reasons. The care of a hospitalized patient with delirium is described as stressful and exhausting [23], especially for nurses trying to identify changes in the cognitive status of patients and at the same time provide safe care [24]. Nurses also reported feelings of discomfort and frustration when providing care for delirium patients. This is arguably due to limited self-confidence in assessing delirium which amplifies the greater dislike of delirium management [25]. Data from studies comparing nurses and doctors, reported similar attitudes and perceptions of delirium [26, 27], highlighting that this is a common and wide-reaching problem, especially in the ICU. In other research, attitudes toward patients with delirium were found to be negative and reports showed patients being viewed as underrated, ignored [28], and considered a “burden”. This can lead to negative outcomes on patients, the hospital staff, and the system of health care because of increased hospitalization, the need for expensive interventions in the event of complications as well as the necessity for long-term care. Most of the current attention focuses on nurses’ knowledge and experiences about delirium in ICU patients and less on nurses’ knowledge and attitudes towards older patients in other hospital wards.

The contribution of this study to the development of knowledge is that it can contribute to improved educational programmes. It can help address delirium knowledge deficits and cultivate positive attitudes towards patients with delirium. The development of a valid and reliable instrument for the measurement of attitudes derived from the study also offers the possibility of assessing nurses’ attitudes further. Nationally, the results are even more important and useful due to the fact that there is no prior study of this subject in the country.

## Methods

### Aim

The study aimed to increase the understanding of delirium care by exploring both knowledge and attitudes of nurses toward patients in acute care hospital wards and the possible association between these two variables.

### Design

This was a cross-sectional study using a descriptive correlational design that was applied to collect data from nurses working in 4 public hospitals in the Republic, between September 2018 and October 2018.

### Sampling

The population under study were all nurses in public hospitals of the country, who worked in acute general wards. They encountered a high number of patients over 65 years of age and therefore high incidences of delirium that is medical, orthopedics, surgical, ED, and ICUs. No exclusion criteria were applied apart from nurses with work experience less than one year. No sampling technique was used, since the questionnaires were administered to all the nurses who worked in the aforementioned wards, thus addressing any potential sampling bias.

### Ethics approval and consent to participate

The study was evaluated and approved by the National Bioethics Committee (SP.2015.01.115), according to the national law, and permission for the collection and management of data was obtained by the Commissioner for Personal Data Protection. Final permission to conduct the research was granted by the Scientific Committee for the Promotion of Research of the Ministry of Health (Fac. No. 5.34.01.7.6E). Also, permission to use the NDK questionnaire was given by the author [29] via email. The participants were informed in writing about the purpose of the study. Completing and returning the questionnaire was considered as consent to participate in the study. The basic principles of ethics in research such as anonymity, informed consent, and confidentiality of data were respected and secured. All participants gave informed consent for the research and the condition that their anonymity was preserved. Participation was voluntary. The data was stored in a manner compliant with data protection regulations. 

### Instruments

For the collection of data, a questionnaire was created and it contained demographic information and two research tools, one for examining the level of knowledge and another for assessing nurse attitudes. The ‘Nurses Knowledge of Delirium’ [29] was used, containing 36 multiple and true/false questions referring to (a) the definition of delirium (b) the tools for delirium identification and (c) the presence of delirium and risk factors leading to delirium development.

The questionnaire was translated into Greek according to the scientific guidelines suggested by the MAPI Research Institute [30], and Medical Outcomes Trust Bulletin [31].

The validity and reliability of the Greek version were tested, including the evaluation of face validity with a group of experts and clinicians and a test-retest analysis. The Cronbach’s alpha coefficient was 0.86, an indication that the questionnaire is highly reliable.

For examining nurses’ attitudes, very few instruments were found focusing specifically on delirium (and none of the publications examined the validity and reliability of the instruments). Therefore, the development and validation of a new tool were deemed necessary. This process consisted of five steps, namely (a) content identification (b) content development (c) content critique (d) the pilot study and (e) field study consisting of psychometric testing of the tool through calculation of the internal consistency and construct validity. This work resulted in the creation of the Attitude Tool of Delirium (ATOD), consisting of 26 questions on a Likert scale of 1–5. The instrument had a Cronbach’s alpha coefficient of 0.89. Factor analysis extracted three factors, corresponding to 56.5% of the variance. These factors are “emotions”, “behavior”, and “beliefs”, corresponding to 37.025%, 12.792%, and 5.652% of the variance respectively. The whole process of the development and validation of the ATOD is presented in a separate publication [32].

### Data analysis

The IBM-SPSS 23 (Statistical Package for Social Sciences) was used for the statistical analysis of the data and the statistical significance was set at *p* < 0.05.

For the questionnaire measuring nurses’ knowledge, the internal consistency was determined using the Kuder-Richardson formula (KR-20) for binary questions (wrong/correct), where values close to 1 are considered satisfactory. The questions that were answered correctly were added up to calculate the level of knowledge of each participant regarding acute confusion / delirium. The percentage of correct answers for each question determined the “Difficulty Index” (DIF) of the question, where high values of DIF showed that the question was easier (i.e., it was answered correctly by more respondents). Moreover, various indexes examined the ability of each question to divide participants into those who knew the subject well and those who did not, for example the “index of divisive power”. A question was considered to have little divisive value when either barely any nurses answered it correctly or when almost everyone answered it correctly. This index was calculated with point-biserial correlations (r_pb_) [33], which measured the correlation between the respondent’s score on a binary (correct/wrong) question and the total score (i.e. the total number of correct answers in the questionnaire on knowledge), indicating if a question has high divisive power (high values of r_pb,_ with *p* < 0.05) or if specific questions need improvement. Another related index was the “Item Discrimination Index” (D.I.), which shows the discriminative ability of the questions to group respondents into “strong” and “weak” based on their knowledge (Hughes, 2003). More specifically, in our study, this index indicated whether the nurses that were expected to answer correctly indeed possessed the relevant knowledge, or whether those who performed poorly on the questionnaire (low scorers) were those that were not expected to have adequate knowledge on the topic. The calculation of the D.I. of a question was as follows: The questionnaires were divided into three groups: group A (1/3 of the questionnaires) included those with the highest score (those who had more than 18 correct answers), group C (also 1/3 of the questionnaires; same sample size as group A) included those with the lowest score - meaning those with less than 13 correct answers - and group B (the remaining 1/3 of the questionnaires) included those with the average score, ranging between 13 and 18 correct answers. From each question, the correct answers of group A and group C were counted. The Discrimination Index of a question was thus calculated using the following formula*:

$$D.I.=\frac{number\;of\;correct\;answer\;in\;Group\;A-number\;of\;correct\;answers\;in\;Group\;C\;}{total\;number\;of\;participants\;in\;Group\;A}$$

*Where Group A is the group with the highest score and Group C is the group with the lowest score.

Generally, a questionnaire item is considered “good” if D.I.>0.40 and 0.30 < DIF < 0.70.

For the questionnaire measuring attitudes, validity and reliability tests were performed [32]. In addition, for all items, mean values and percentages in each category indicated positive, negative, or neutral attitudes.

Significant relationships between nurses’ knowledge and attitudes were explored with Pearson correlation coefficients, where a relation is considered significant if *p* < 0.05, with the corresponding sign (positive or negative). Finally, to identify differences in the level of knowledge and attitudes according to nurses’ socio-demographics, various statistical tests were done for all the scales and subscales. These included independent samples t-tests (to examine gender differences in the scales/subscales of knowledge and attitudes), ANOVA tests (to examine differences in knowledge and attitudes between hospitals, job positions, wards, education levels) and chi-square tests (for the dichotomous item 1.1 of the knowledge scale). Regression analysis was also used, to examine the effect of knowledge and personal characteristics (when entered simultaneously in the model) on attitudes (dependent variable). It is noted that no missing data existed for the attitudes’ questionnaire, whereas the missing data (around 1% of the sample) and the “Do not know” answers that existed in the knowledge questionnaire were classified as “wrong” answers.

## Results

### Profile of the participants

**Fig. 1:**
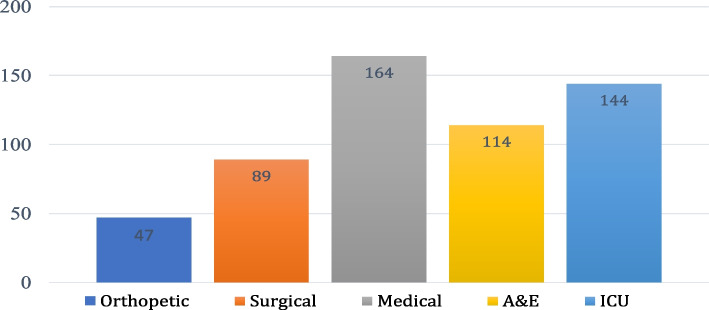
Participants- Clinical Setting (*N*=558)

The final questionnaire was given to 835 nurses working in departments with increased incidence of delirium and older patients such as orthopaedic, acute medical and surgical departments, intensive care units, and accident and emergency departments, in 4 Public Hospitals in the country. As shown in Fig. 1 below, most of the participants worked in ICU and medical departments (Fig. 1). Response rate was 67% and the final sample size was 558 nurses.

Age ranged from 21 to 65 years, with an average of 35.8 years and a standard deviation of 8.2 years. In terms of total experience, it was from 1 to 35 years, with an average of 13.2 years and a standard deviation of 8.2 years. The experience in the department ranged from 1 to 27 years with an average of 6.8 years and a standard deviation of 6.0 years. Most of the participants were women (312, 56.1%), had a bachelor’s degree (552, 99%), were graduate nurses without postgraduate studies (323, 58%), while 149 nurses (26.8%) reported postgraduate studies. All nurses were registered nurses who were employed in the Public Hospital as permanent staff or under a contract of indefinite duration.

### The level of knowledge

The questionnaire consists of 36 questions. The analysis was performed on the overall scale but also for the three subscales – the three parts of the questionnaire: 1- definition of delirium (question 1.1), 2- tools for delirium recognition (questions 1.2–1.8), and 3- the presence of delirium and risk factors for developing delirium (questions 1.9–1.36).

The Kuder-Richardson 20 values were satisfactory, indicating overall high internal consistency for the knowledge questionnaire (KR-20 = 0.742 for the total scale, 0.722 for questions 1.9–1.36, and 0.400 for questions 1.2–1.8, where question 1.2 was the least reliable). In 25 out of 36 delirium-oriented questions, most nurses answered incorrectly or declared that they did not know the answer. The correct answer average was as high as 42.2%. Only 25.6% of the participants scored below 18, which is the average.

Table 1 below shows the results from the descriptive statistics on the number of correct responses given by participants for each subscale of the questionnaire and on the total scale (36 questions) (Table 1). Results show that 3 out of 4 participants (75%), answered correctly on less than 19 of the 36 questions.

**Table 1:** Descriptive statistics for the number of correct answers per category

Category of questionnaire questions (subscales)	Mean	Standard Deviation	Minimum value (Number of correct questions)	Maximum value (Number of correct questions)	P 25 (Number of correct questions)	Median (Number of correct questions)	P75 (Number of correct questions)
**Tools for Delirium Identification (Q2-Q8)** **(7 questions)**	2.90	1.33	0	6	2	3	4
**Presence of Delirium and risk factors leading to Delirium development (Q9-Q36)** **(28 questions)**	11.88	4.54	0	25	9	12	15
**Total Scale (Q1-Q36)** **(36 questions)**	15.14	5.28	1	30	11	15	19

In Table 2 below, a more detailed analysis of each question of the knowledge instrument is presented, showing the Difficulty Index (DIF) of each question.

**Table 2:** The level of Knowledge per question (Difficulty Index (%)

Question (*from NDK questionnaire* [29] ) No. Participants *N* = 558 Correct Αnswers (Ν) /558	Difficulty Index, DIF, %
**Definition**	
1. Which of the following groups of symptoms best describes or defines delirium? (*Ν* = 212)	38%
**Tools for Delirium Identification**	
2. Mini-Mental State Examination (MMSE) (*Ν* = 87)	15.6%
3. Glasgow Coma Scale (GCS) (*Ν* = 389)	69.7%
4. Delirium Rating Scale (DRS) (*Ν* = 290)	52.0%
5. Alcohol Withdrawal Scale (AWS) (*Ν* = 26)	4.7%
6. Confusion Assessment Method (CAM) (*Ν* = 125)	22.4%
7. Beck’s Depression Inventory (*Ν* = 405)	72.6%
8. Braden Scale (*Ν* = 299)	53.6%
**Presence of Delirium and risk factors**^**†**^**leading to delirium development**	
9. Fluctuation between orientation and disorientation is not typical of delirium (*Ν* = 225)	40.3%
10. Symptoms of depression may mimic delirium (*Ν* = 182)	32.6%
11. Treatment for delirium always includes sedation (*Ν* = 299)	53.6%
12. Patients never remember episodes of delirium (*Ν* = 150)	26.9%
13. A Mini-Mental Status Examination (MMSE) is the best way to diagnose delirium (*Ν* = 108)	19.4%
14^†^. A patient having a repair of a fractured neck of femur has the same risk for delirium as a patient having an elective hip replacement (*Ν* = 162)	29.0%
15. Delirium never lasts for more than a few hours *Ν* = 274	49.1%
16^†^. The risk for delirium increases with age (*Ν* = 296)	53.0%
17^†^. A patient with impaired vision is at increased risk of delirium (*Ν* = 181)	32.4%
18^†^. The greater the number of medications a patient is taking, the greater their risk of delirium (*Ν* = 262)	47.0%
19^†^. A urinary catheter in situ reduces the risk of delirium (*Ν* = 364)	65.2%
20^†^. Gender has no effect on the development of delirium (*Ν* = 133)	23.8%
21^†^. Poor nutrition increases the risk of delirium (*Ν* = 241)	43.1%
22^†^. Dementia is the greatest risk factor for delirium (*Ν* = 263)	47.1%
23^†^. Males are more at risk for delirium than females (*Ν* = 110)	19.7%
24^†^. Diabetes is a high-risk factor for delirium (*Ν* = 146)	26.2%
25^†^. Dehydration can be a risk factor for delirium (*Ν* = 368)	65.9%
26^†^. Hearing impairment increases the risk of delirium (*Ν* = 216)	38.7%
27^†^. Obesity is a risk factor for delirium (*Ν* = 248)	44.4%
28. A patient who is lethargic and difficult to rouse does not have a delirium (*Ν* = 251)	45.0%
29. Patients with delirium are always physically and/or verbally aggressive (*Ν* = 281)	50.4%
30. Delirium is generally caused by alcohol withdrawal (*Ν* = 266)	47.7%
31^†^. Patients with delirium have a higher mortality rate (*Ν* = 178)	31.9%
32^†^. A family history of dementia predisposes a patient to delirium (*Ν* = 131)	23.5%
33. Behavioural changes in the course of the day are typical of delirium (*Ν* = 224)	40.1%
34. A patient with delirium is likely to be easily distracted and/or have difficulty following a conversation (*Ν* = 388)	69.5%
35. Patients with delirium will often experience perceptual disturbances (*Ν* = 385)	69.0%
36. Altered sleep/wake cycle may be a symptom of delirium (*Ν* = 301)	53.9%

In total, out of 36 questions, only 12 of those had a correct response ratio higher than 50%, i.e. only 1/3 of the questions had a high value of DIF and could be considered easy. The easiest question for respondents was question 1.7 (Assessment Tool - Depression Diagnosis, the Beck’s Depression Recording Scale) which 72.6% of participants answered correctly. The question with the lowest DIF (in other words, the most difficult question) was question 1.5, which referred to the tool for assessing delirium and alcohol use and abuse - Alcohol core Deprivation Scale - AWS, while there were several other questions with a low DIF, even below the 0.30 limit. In general, it seems that in this questionnaire there were no questions that can be considered very easy (DIF > 0.9) (without practical value or ability to separate nurses). However, there were some that can be considered as very difficult for the participants (DIF < 0.3) (such as questions 1.2, 1.5, 1.6, 1.12, 1.13, 1.14, 1.20, 1.23, 1.24, 1.32).

In addition, the index of divisive power was calculated. Most questions had high divisive power (high values of r_pb_ with *p* < 0.05), which shows that either they were answered correctly by the participants who received high total scores in the questionnaire, or they were answered wrongly by nurses who had low total scores. The only exceptions were questions 1.2, 1.5, 1.24 and 1.32 (r_pb_<0.1, *p* > 0.05). Their low correlations showed either that the participants had correct answers to the other questions but not to those, or participants did not understand the specific questions but had a high total questionnaire score. Therefore, these questions would need improvement or require attention as to the knowledge regarding their content. As seen before, the same questions gave the most trouble to participants, since they also had a low DIF.

Another approach to the discriminative ability of the questions was the Item Discrimination Index (D.I.), which indicated if the nurses that were expected to answer correctly indeed had the knowledge, or that those who performed poorly were not expected to adequately possess the subject matter. Several questions had a Discrimination Index below 0.4, while questions 1.5 and 1.32 had their D.I. almost being zero. So, combining all the above results confirms that especially questions 1.5 and 1.32 need attention because they were particularly difficult for the participants.

### Nurses’ attitudes

As regards the questionnaire for the investigation of attitudes, the 5-point Likert scale was dichotomized into negative (1–2) and positive (4–5), while 3 was considered neutral. The results showed that in 14 questions more than 50% of the participants had a negative attitude and only 9% declared a positive attitude. The highest negative results were found in questions 2.19, 2.1, and 2.14. It is also interesting that a high percentage of the participants (around 1/3 of participants) selected a neutral position in many questions (2.1. 2.2, 2.15, 2.17–2.19, 2.21–2.23). The results are shown in Table 3:

**Table 3:** Nurses’ attitudes results, mean and standard deviation (Range = 1–5)

Question	Negative attitude % (1–2)	Positive attitude % (4–5)	Neutral attitude % ( 3 )	Mean	Standard Deviation
**Emotions** **1. I feel comfortable caring for a patient with acute confusion/delirium** **2. I trust myself that it implements the appropriate interventions for a patient with OS/P** **3. I would like to have continuous guidance regarding the care of patients with acute confusion/delirium** **8. I try to manage the situation to help the patient overcome the crisis** **9. I continue to care about the patient’s health as much as I was interested in before the arousal crisis** **Subscale**	57.3% 39.5% 7.1% 17.9% 13.8%	12.2% 31.1% 77.8% 66.7% 67.7%	30.5% 29.4% 15.1% 15.6% 18.5%	2.27 2.73 4.02 3.64 3.77 **3.29**	1.02 1.15 0.91 0.95 1.00 **0.64**
**Behavior** **4. When a patient experiences acute confusion/delirium, I take it into account and take it seriously.** **5. I try to better understand the patient with acute confusion/delirium.** **6. I avoid patients with such problems during my shift.** **7. I treat patients with acute confusion/delirium with patience** **Subscale**	18.7% 20.1% 30.8% 19.6%	66.8% 61.4% 47.7% 57.8%	14.5% 18.5% 21.5% 22.6%	3.70 3.55 3.24 3.54 **3.51**	1.04 1.01 1.28 1.02 **0.90**
**Beliefs** **10. I find the acute confusion/beaches. is a phenomenon that occurs mainly in the elderly.** **11. Polite speech can calm a patient with acute confusion/delirium.** **12. I consider the condition of the patient with acute confusion/delirium to be very serious.** **13. When a patient tries to beat me, I try to tie him.** **14. When an elderly patient is in acute confusion, I suspect that there may be some other problem.** **15. The best indication of the presence of acute confusion/ delirium in patients is disorientation** **16. Colleagues are willing to help when I have questions about a patient with acute confusion/delirium** **17. I clearly understand the reason for the patient’s agitation** **18. I don’t feel comfortable hospitalizing a patient with acute confusion/delirium.** **19. I believe that the use of physical limitations in patients with acute confusion/delirium is necessary** **20. I believe that acute confusion/delirium is a phenomenon that occurs in different departments of a hospital and not only in ICU.** **21. I find acute delirium confusion is a syndrome that is underdiagnosed.** **22. I find that acute confusion/delirium can be largely prevented.** **23. I find acute confusion/delirium to be a manageable situation.** **Subscale**	34.8% 25.8% 16.1% 39.0% 62.9% 23.5% 29.2% 35.3% 47.7% 57.0% 12.6% 8.8% 20.7% 27.5%	39.6% 47.0% 60.8% 33.4% 15.1% 46.0% 49.6% 31.9% 23.3% 10.0% 72.4% 59.1% 41.8% 41.0%	25.6% 27.2% 23.1% 27.6% 22.0% 30.5% 21.1% 32.8% 29.0% 33.0% 14.9% 32.1% 37.5% 31.5%	3.03 3.31 3.59 2.92 2.42 3.22 3.20 2.92 2.59 2.38 3.83 3.60 3.25 3.13 **3.10**	1.08 1.04 0.98 1.11 0.94 0.97 1.09 1.01 1.16 0.91 1.00 0.84 0.91 0.97 **0.43**
**Total scale**				**3.21**	**0.50**

### The relation between knowledge and attitudes

Pearson’s correlation coefficients examined all the relationships between the scales of knowledge and attitudes, as well as within each scale (i.e. between the total scale and its subscales).

First, regarding the knowledge questionnaire, the correlations between the total scale and its sub-scales (i.e., subscale of items 1.2–1.8, subscale of items 1.9–1.36 and item 1.1 (dichotomous; point-biserial correlation)) were significant positive, showing that nurses who knew the correct answers to one part of the questionnaire knew them in other parts as well. Second, regarding the “attitudes” questionnaire, the significant positive relationships that were found between the overall scale and its three factors (behavior, emotion, perception) indicated that nurses who had a positive attitude in one factor had a positive attitude in the other factors as well.

Third, one of the research questions was whether there was a relationship between the knowledge and attitudes of the participants. Pearson’s correlation coefficients for the relationships between the scales of knowledge (total scale and subscales) and attitudes (total scale and three factors), showed a significant positive relationship between knowledge and attitudes (all scales and subscales). Therefore, the important result that emerges is that the better knowledge a nurse has about delirium, the more positive attitude he/she has towards patients with delirium. Table 4 shows the results regarding the relations between the scales/subscales of knowledge and attitudes.

**Table 4:** The relation of knowledge and attitudes (Pearson correlation coefficients)

Questions	Knowledge 1.2–1.8	Knowledge 1.9–1.36	Knowledge 1.1–1.36	Attitudes Total	Attitudes Emotion	Attitudes Behavior	Attitudes Perception
**Knowledge(1.1)** **Definition of Delirium**	0.21^**^ < 0.001	0.26^**^ < 0.001	0.37^**^ < 0.001	0.35^**^ < 0.001	0.29^**^ < 0.001	0.31^**^ < 0.001	0. 33^**^ < 0.001
**Knowledge(1.2–1.8)** **Tools for Delirium** **Identification**		0.36^**^	0.55^**^	0.48^**^	0.42^**^	0.44^**^	0.44^**^
		< 0.001	< 0.001	< 0.001	< 0.001	< 0.001	< 0.001
**Knowledge(1.9–1.36)** **Presence of Delirium** **and Risk factors**			0.96^**^	0.48^**^	0.40^**^	0.41^**^	0.47^**^
			< 0.001	< 0.001	< 0.001	< 0.001	< 0.001
**Knowledge** **Total**				0.56^**^	0.47^**^	0.48^**^	0.54^**^
				**< 0.001**	< 0.001	< 0.001	< 0.001
**Attitudes** **Total**					0.87^**^	0.89^**^	0.93^**^
					< 0.001	< 0.001	< 0.001
**Attitudes** **Emotion**						0.75 ^**^	0.69^**^
						< 0.001	< 0.001
**Attitudes** **Behavior**							0.71 ^**^
							< 0.001

### Differences in knowledge and attitudes according to personal characteristics

First, we examined which personal characteristics affect the level of knowledge of nurses about acute confusion / delirium. Statistically significant gender differences for the knowledge subscale of items 1.2 to 1.8 were found, where women had better knowledge than men in these specific questions (mean = 3.0 and 2.8 respectively; *p* = 0.030), as well as for item 1.1 (definition of delirium) (*p* = 0.015), where 42% of women answered it correctly as opposed to 32% of men. For the other scales, there were no differences (for 1.9–1.36 *p* = 0.745, for 1.1–1.36 *p* = 0.351). No significant differences in nurses’ knowledge were found between hospitals (p > 0.05) or job positions (*p* > 0.05). The ANOVA test for “ward” showed significant differences (*p* < 0.001 for the total scale; *p* = 0.003 for 1.2–1.8; *p* = 0.001 for 1.9–1.36). More specifically, Tukey’s post-hoc tests showed that nurses working in ICUs had better knowledge compared to all the other wards. Similarly, in terms of education level, significant differences were identified (*p* < 0.05 for scales and subscales), where it was evident from post-hoc tests, that the more educated nurses were, the better knowledge they had, especially if nurses had a master’s degree.

Second, concerning nurses’ attitudes, the only demographic variable that showed significant differences was “Ward”, where nurses in the ICU reported more positive attitudes (in perception) (*p* = 0.026), compared to nurses working in other wards (especially medical wards).

Finally, regression analysis additionally showed that when knowledge and personal characteristics were entered simultaneously in the model (i.e., adjusting for the confounding effect of socio-demographics), only knowledge appears to affect the attitudes of nurses (*p* < 0.001), which verifies the previous findings of a significant positive correlation between knowledge and attitudes.

## Discussion

Observing nurses in the public hospital sector, overall, their knowledge about acute confusion / delirium is insufficient. The average overall correct answer rate was 42.2% (15/36) and is similar to that of the equivalent study conducted in Australia by the creators of the tool (53.14%) (Hare et .al., 2008). Similar results were noted by other studies conducted to investigate the level of nurses’ knowledge of delirium [0, 35].

Low knowledge levels were also observed in a systematic review of 10 studies where it was found that nurses did not recognize delirium in older patients [36], as well as in studies involving hospitalized older patients [37, 38]. Similar results were found in a study of the level of delirium knowledge of nurses in Greece, from a sample of 108 nurses working in medical and surgical units in two general hospitals in Athens and three provincial hospitals. In this study, nurses reported that they were not trained and therefore unqualified in assessing patients with delirium symptoms. This finding is justified by the respondents’ reply that 67% had learned nothing about delirium [39].

The low knowledge level of nurses in our research can be partly explained by the limited focus on delirium during basic nursing education. In examining the curriculums of the five Universities in the country offering nursing studies, it was found that confusion and delirium are mentioned briefly in some sections of neurology subjects (e.g., consciousness), in psychiatric nursing modules regarding alcoholic delirium and a reference to the section on gerontological nursing, but without individual focus on delirium in hospitalized patients. The curriculums that had been examined cover the syllabi of the last two decades. We examined the titles and course descriptions of the nursing programs to see whether there were individual modules or sections on delirium. Similarly, the subject of delirium is not a priority topic in the incumbent nursing education programmes that are compulsory for renewing licences according to national law.

Nurses’ knowledge of acute confusion / delirium is largely empirical rather than theoretically based. This view is evidenced by the limited correct answers to questions concerning the risk factors of delirium development in contrast with the presence of delirium. Knowledge of the presence of delirium could be acquired empirically, while questions related to evaluation tools, - something that nurses have never use - require theoretical knowledge and experience in using the tool, something which they predominantly did not possess. In the study only 22.4% answered correctly in the question about the use of the Confusion Assessment Method (CAM) tool, which is considered the gold standard for assessing delirium [40].

The findings of this study show that the biggest gap in knowledge concerning acute confusion / delirium is the theoretical aspect (definition, assessment scales, symptoms, risk factors of development of delirium, etc.) which cannot be fulfilled with vocational experience. This should be taken into consideration when designing the curriculum content of future training and educational programmes. Educators should focus on the provision of mainly theoretical knowledge, which can enhance the existing knowledge of nurses acquired from practical experience. The implementation of such training programmes resulted in a significant improvement in the level of knowledge of delirium in nurses [41, 42], using the NKDQ tool.

Concerning nurses’ attitudes toward delirium, the results showed that most nurses had a negative attitude, supporting previous studies [25, 43]. A higher percentage of negative attitudes involved the use of physical restraints on the basis that they were necessary for the patient’s care (90%). Indeed, the literature confirms that conditions such as anxiety, agitation, confusion, hallucinations, aggression and changes in the severity of the disease and its unpredictability, contribute to further negative attitudes. These include the feeling of burden [44], debilitation [23] and lack of confidence when managing arousal and delirium [43]. The results also reveal that nurses do not view delirium as a serious condition and therefore it is underestimated and not prioritized in their work [45, 46]. Although several nurses reported instances of evaluating delirium, very few of them used assessment tools in the identification process [47].

The assumption that there is a relationship between knowledge and attitudes of nurses was examined with the correlation coefficient Pearson and it was found that the better the knowledge, the more positive attitude towards delirium, agreeing with the literature, regardless of the tools used [27]. However, studies are showing that even if a strong emphasis is placed on improving knowledge, this does not mean that nurses will develop a positive attitude towards the older people or patients with cognitive decline [48].

This means that the hypothesis that increasing knowledge can lead to better care raises concerns about the methodological approach of these two concepts. Intervention strategies on improving the knowledge of nurses have yet to tackle the complexity of changing attitudes. However, according to social learning theory, environmental and cognitive factors inherently influence human learning and behaviour. For example, observing, modelling, and imitating the behaviors and emotional reactions of others can stimulate changes in attitudes [49]. The scientific approach to behaviour change needs clear and well-defined guidelines for identifying the ‘active ingredients’ and for designing, evaluating and reporting interventions [50]. Investigating the attitudes of nurses towards the care of delirium through qualitative research will identify the reasons for these attitudes and identify the educational needs of nurses in terms of care management.

### Limitations of the study

This study was conducted only in public hospitals in the country. The participation of nurses from private hospitals was not possible for this inquiry and the interpretation and inferences of the results should be done carefully.

## Conclusions

Based on the results of the study, it seems that the elaboration of continuous educational programmes is needed to improve the level of knowledge of nurses on acute confusion / delirium which could potentially improve their attitude towards delirium. These programmes should focus mainly on providing theoretical knowledge (definition, scales, symptoms, risk factors for delirium, etc.) – as demonstrated by the questionnaire answers – in order to boost the existing empirical knowledge of nurses.

Monitoring and evaluating nurses’ knowledge and attitudes toward patients with delirium could be the first step to detecting the gaps in treating older delirium sufferers effectively. This is useful towards a greater understanding of care management and its challenges. It is also a reminder to medical educators about the importance of ongoing education in hospitals.

Nursing managers and policymakers also need to formulate hospital policies to improve the quality of care provided for older people. The introduction of clinical guidelines, protocols, delirium care bundles and assessment scales could reduce the duration of hospitalization but also the cost of the provided care.

## Data Availability

The datasets used and/or analyzed during the current study are available from the corresponding author on reasonable request.

## References

[1] National Institute for Health and Care Excellence (NICE). Delirium: prevention, diagnosis and management Clinical guideline.2010; ISBN:978-1-4731-2992-4. https://www.nice.org.uk/guidance/cg103.

[2] American Psychiatric Association. Diagnostic and statistical manual of mental disorders. 5th edition. Arlington VA; 2013. ISBN: 978-0-89042-555-8. 10.1176/appi.books.9780890425596.

[3] Inouye SK, Westendorp RGJ, Saczynski JS (2014). Delirium in elderly people. Lancet.

[4] Hshieh TT, Inouye SK, Oh ES (2020). Delirium in the Elderly. Clin Geriatr Med.

[5] Boettger S, Zipser CM, Bode L, Spiller T, Deuel J, Osterhoff G (2021). The prevalence rates and adversities of delirium: too common and disadvantageous. Palliat Support Care.

[6] OECD (2012). Health Data 2012.

[7] Zipser CM, Deuel JW, Held JPO, Ernst J, Schubert M, Weller M, et al. Economic impact of Poststroke Delirium and Associated Risk factors: findings from a prospective cohort study. Stroke. 2021;52(10):3325-3334. 10.1161/STROKEAHA.120.033005. 10.1161/STROKEAHA.120.033005 34233463

[8] Kinchin I, Mitchell E, Agar M, Trépel D. The economic cost of delirium: A systematic review and quality assessment. 2021. 10.1002/alz.12262. 10.1002/alz.12262 33480183

[9] Caplan GA, Teodorczuk A, Streatfeild J, Agar MR (2020). The financial and social costs of delirium. Eur Geriatr Med.

[10] Boustani M, Rudolph J, Shaughnessy M, Gruber-Baldini A, Alici Y, Arora RC (2014). The DSM-5 criteria, level of arousal and delirium diagnosis: inclusiveness is safer. BMC Med.

[11] Rood P, Huisman - de Waal G, Vermeulen H, Schoonhoven L, Pickkers P, van den Boogaard M (2018). Effect of organisational factors on the variation in incidence of delirium in intensive care unit patients: a systematic review and meta-regression analysis. Aust Crit Care.

[12] Krewulak KD, Stelfox HT, Leigh JP, Wesley Ely E, Fiest KM (2018). Incidence and prevalence of Delirium Subtypes in an adult ICU: a systematic review and Meta-analysis. Crit Care Med.

[13] Gehrke S, Bode L, Seiler A, Ernst J, Von Känel R, Boettger S (2021). The prevalence rates and sequelae of delirium at age older than 90 years. Palliat Support Care.

[14] Singler K, Thomas C (2017). HELP - hospital elder life program - multimodal delirium prevention in elderly patients. Internist (Berl).

[15] Robinson TN, Eiseman B (2008). Postoperative delirium in the elderly: diagnosis and management. Clin Interv Aging.

[16] Bush SH, Tierney S, Peter •, Lawlor G (2017). Clinical Assessment and Management of Delirium in the Palliative Care setting. Drugs.

[17] Mori S, Takeda JRT, Carrara FSA, Cohrs CR, Zanei SSV, Whitaker IY (2016). Incidence and factors related to delirium in an intensive care unit. Rev Esc Enferm USP.

[18] Mattison MLP, Delirium (2020). Ann Intern Med.

[19] Ahmed S, Leurent B, Sampson EL (2014). Risk factors for incident delirium among older people in acute hospital medical units: a systematic review and meta-analysis. Age Ageing.

[20] Ticinesi A, Cerundolo N, Parise A, Nouvenne A, Prati B, Guerra A (2020). Delirium in COVID-19: epidemiology and clinical correlations in a large group of patients admitted to an academic hospital. Aging Clin Exp Res.

[21] Carin-Levy G, Nicol K, van Wijck F, Mead G, McVittie C (2021). Identifying and responding to Delirium in Acute Stroke:clinical team members’ understandings. Qual Health Res.

[22] Lee G, Roh YS (2021). Knowledge, barriers, and training needs of nurses working in delirium care. Nurs Crit Care.

[23] LeBlanc A, Bourbonnais FF, Harrison D, Tousignant K (2018). The experience of intensive care nurses caring for patients with delirium: a phenomenological study. Intensive Crit Care Nurs.

[24] Brooke J, Manneh C. Caring for a patient with delirium in an acute hospital: The lived experience of cardiology, elderly care, renal, and respiratory nurses. Int J Nurs Pract. 2018;24(4):e12643. 10.1111/ijn.12643. 10.1111/ijn.12643 29532553

[25] Zamoscik K, Godbold R, Freeman P (2017). Intensive care nurses’ experiences and perceptions of delirium and delirium care. Intensive Crit care Nurs.

[26] Selim AA, Wesley Ely E (2017). Delirium the under-recognised syndrome: survey of healthcare professionals’ awareness and practice in the intensive care units. J Clin Nurs.

[27] Nydahl P, Dewes M, Dubb R, Hermes C, Kaltwasser A, Krotsetis S (2018). Survey among critical care nurses and physicians about delirium management. Nurs Crit Care.

[28] Inouye SK (2018). Delirium—A Framework to improve Acute Care for older persons. J Am Geriatr Soc.

[29] Hare M, Wynaden D, McGowan S, Landsborough I, Speed G. A questionnaire to determine nurses' knowledge of delirium and its risk factors. Contemp Nurse. 2008;29(1):23-31. 10.5172/conu.673.29.1.23. 10.5172/conu.673.29.1.23 18844539

[30] MAPI Research Institute. The MAPI Linguistic Validation Process. 2002. http://www.mapi-research-inst.com/lvprocess.asp. Accessed 1 May 2022.

[31] Trust MO. Trust introduces new translation criteria. Medical Outcomes Trust Bulletin. 1997;5:3–4. http://www.outcomes-trust.org/bulletin/0797blltn.htm. Accessed 1 May 2022.

[32] Papastavrou E, Papaioannou M, Evripidou M, Tsangari H, Kouta C, Merkouris A (2019). Development of a Tool for the Assessment of Nurses’ Attitudes Toward Delirium. J Nurs Meas..

[33] Glass GV, Hopkins KD. Statistical Methods in Education and Psychology. 3rd edition. Boston: Allyn & Bacon; 1995. ISBN: 978-0-205-14212-5.

[34] Akrour R, Verloo H (2017). An observational study of communityhealth care nurses&rsquo; knowledge about delirium. Nurs Res Rev..

[35] Hickin SL, White S, Knopp-Sihota J (2017). Nurses’ knowledge and perception of delirium screening and assessment in the intensive care unit: long-term effectiveness of an education-based knowledge translation intervention. Intensive Crit Care Nurs.

[36] Steis MR, Fick DM. Are nurses recognizing delirium? A systematic review. Journal of Gerontological Nursing. 2008;34(9):40-48. 10.3928/00989134-20080901-12. 10.3928/00989134-20080901-12 18795564

[37] El Hussein M, Hirst S, Salyers V (2015). Factors that contribute to underrecognition of delirium by registered nurses in acute care settings: a scoping review of the literature to explain this phenomenon. J Clin Nurs.

[38] van Velthuijsen EL, Zwakhalen SMG, Mulder WJ, Verhey FRJ, Kempen GIJM (2018). Detection and management of hyperactive and hypoactive delirium in older patients during hospitalization: a retrospective cohort study evaluating daily practice. Int J Geriatr Psychiatry.

[39] OC K (2012). Nurses’ knowledge of care for patients with delirium. Rostrum of Asclepius.

[40] Quispel-Aggenbach DWP, Holtman GA, Zwartjes HAHT, Zuidema SU, Luijendijk HJ. Attention, arousal and other rapid bedside screening instruments for delirium in older patients: a systematic review of test accuracy studies. Age Ageing. 2018 Sep 1;47(5):644–653. doi: 10.1093/ageing/afy058. PMID: 29697753. 10.1093/ageing/afy058 29697753

[41] Speed G (2015). The impact of a delirium educational intervention with intensive care unit nurses. Clin Nurse Spec.

[42] Van De Steeg L, Jkema RI, Wagner C, Langelaan M (2015). The effect of an e-learning course on nursing staff’s knowledge of delirium: a before-and-after study. BMC Med Educ.

[43] Tsang JLY, Ross K, Miller F, Maximous R, Yung P, Marshall C, et al. Qualitative descriptive study to explore nurses’ perceptions and experience on pain, agitation and delirium management in a community intensive care unit. BMJ Open. 2019;9(4):e024328. Published 2019 Apr 4. 10.1136/bmjopen-2018-024328. 10.1136/bmjopen-2018-024328 PMC6500293 30948568

[44] Schmitt EM, Gallagher J, Albuquerque A, Tabloski P, Lee HJ, Gleason L (2017). Perspectives on the Delirium experience and its Burden: common themes among older patients, their family caregivers, and nurses. Gerontologist.

[45] Emme C (2020). “It should not be that difficult to manage a condition that is so frequent”: a qualitative study on hospital nurses’ experience of delirium guidelines. J Clin Nurs.

[46] Biyabanaki F, Arab M, Dehghan M (2020). Iranian Nurses Perception and Practices for Delirium Assessment in Intensive Care Units. Indian J Crit Care Med.

[47] Özsaban A, Acaroglu R (2016). Delirium assessment in intensive care units: practices and perceptions of turkish nurses. Nurs Crit Care.

[48] Hammar LM, Holmström IK, Skoglund K, Meranius MS, Sundler AJ (2017). The care of and communication with older people from the perspective of student nurses. A mixed method study. Nurse Educ Today.

[49] Bandura A (1977). Social learning theory. Englewood Cliffs.

[50] Michie S, Johnston M (2012). Theories and techniques of behaviour change: developing a cumulative science of behaviour change. Health Psychol Rev.

